# Exercise self-efficacy and the relation with physical behavior and physical capacity in wheelchair-dependent persons with subacute spinal cord injury

**DOI:** 10.1186/s12984-015-0099-0

**Published:** 2015-11-19

**Authors:** Carla F.J. Nooijen, Marcel W.M. Post, Annemie L. Spooren, Linda J. Valent, Rogier Broeksteeg, Tebbe A. Sluis, Henk J. Stam, Act-Active Research Group, Rita J.G. van den Berg-Emons

**Affiliations:** Department of Rehabilitation Medicine, Erasmus MC University Medical Center Rotterdam, P.O. Box 2040, 3000 CA Rotterdam, The Netherlands; Brain Center Rudolf Magnus and Center of Excellence for Rehabilitation Medicine, University Medical Center Utrecht and De Hoogstraat Rehabilitation, Utrecht, The Netherlands; Department of Rehabilitation Medicine, Center for Rehabilitation, University of Groningen, Groningen, The Netherlands; University Medical Center Groningen, Groningen, The Netherlands; Adelante Center of Expertise in Rehabilitation and Audiology, Hoensbroek, The Netherlands; CAPHRI School for Public Health and Primary Care, Department of Rehabilitation Medicine, Maastricht University, Maastricht, Netherlands; Heliomare Rehabilitation Center, Wijk aan Zee, The Netherlands; Rijndam Rehabilitation Institute, Rotterdam, The Netherlands

**Keywords:** Spinal cord injuries, Self-efficacy, Exercise, Physical activity, Physical capacity

## Abstract

**Background:**

Since physical activity and exercise levels are known to be generally low in persons with spinal cord injury (SCI), there seems to be a need for intervention. Exercise self-efficacy (ESE), the confidence persons have in their ability to be physically active and exercise, is an important and modifiable predictor of physical behavior. The goal of this study was to 1) describe ESE in persons with subacute SCI, 2) to assess ESE in subgroups based on demographic and lesion characteristics, and 3) to explore the relation between ESE and physical behavior and physical capacity.

**Methods:**

Thirthy-seven persons with subacute SCI who are wheelchair dependent participated. Participants completed the Exercise Self-Efficacy Scale. We recorded age and lesion characteristics, measured physical behavior (physical activity, motility and sedentary day time, *n* = 35) with an accelerometer-based activity monitor and measured physical capacity (peak power output, *n* = 28 and peak oxygen uptake, *n* = 24) during a maximal hand-cycling test. Measurements were performed 2 months prior to discharge from inpatient rehabilitation. Mann-Whitney tests were used to test for differences between subgroups based on age and lesion characteristics and spearman correlations were used to assess the relation between ESE and physical activity and physical capacity.

**Results:**

Persons with tetraplegia had lower ESE compared to persons with paraplegia (Z = −1.93, *p* = 0.05). No differences in ESE were found between subgroups based on age and motor completeness of the lesion. In persons with paraplegia, ESE was positively related to peak power output (*ρ* = 0.58, *p* = 0.02). The relation of ESE with wheeled physical activity was *ρ* = 0.36, *p* = 0.09.

**Conclusions:**

In persons with SCI who are dependent on a manual wheelchair, lesion level when categorized as paraplegic and tetraplegic affected ESE whereas age categories and completeness categories did not. Persons with tetraplegia were found to have lower confidence with regard to physical activity and exercise indicating that this subgroup can benefit from extra attention in the promotion of physical activity and exercise. In persons with paraplegia, ESE seemed to be lower in persons with less peak power output and less daily physical activity.

## Background

Daily physical activity and exercise are known to reduce the risk of cardiovascular disease, prevent or reduce secondary conditions, and improve quality of life for persons with spinal cord injury (SCI) [1–5]. However, physical activity and exercise levels are known to be generally low in persons with SCI in the chronic phase [6, 7]. In addition to maintaining sufficient physical activity, interposing of breaks in sedentary day time is another independent aspect of physical behavior that is thought to be important for optimal health [8, 9]. Sedentary time is not just the counterpart of physical activity. It has been defined as “any waking sitting or lying behavior with low energy expenditure” [10]. More physical activity and exercise is known to reduce the risk of cardiovascular disease, prevent or reduce secondary conditions, and improve physical fitness and quality of life in persons with SCI [2, 3]. Therefore, promoting an active lifestyle in persons with SCI is important.

Exercise self-efficacy (ESE), the confidence persons have in their ability to exercise [11], is an important and modifiable predictor of physical activity and exercise behavior [12]. Persons with higher ESE are more likely to be physically active and to exercise [13]. During the first months after SCI, persons establish a new routine in their wheelchair and therefore this period might be critical to introduce and encourage new habits that incorporate physical activity and exercise and limit sedentary day time. By helping persons to increase ESE, practitioners may be more successful in achieving higher levels of physical activity and exercise [13]. A previous study showed that a behavioral intervention targeting ESE was effective in increasing the frequency of physical activity and exercise among persons with SCI in the chronic phase [14]. However, to our knowledge this has not been studied in persons with subacute SCI. Furthermore, it is unknown what determines ESE in persons with subacute SCI. Among able-bodied adolescents [15] and older adults [16], men show higher ESE compared to women. Among persons with coronary heart disease, age is inversely associated with ESE [17]. Furthermore, ESE has been related to aerobic capacity among middle-aged adults [18].

The goal of this study was to 1) describe ESE in persons with subacute SCI, 2) to assess ESE in subgroups based on demographic and lesion characteristics, and 3) to explore the relation between ESE and physical behavior and physical capacity. We hypothesized that ESE was lower among females, older persons, persons with tetraplegia and among those with motor complete lesions. Furthermore, we expected ESE to be lower among persons with lower daily physical activity levels, with more sedentary time, and with lower physical capacity. This study may assist in identifying subgroups of persons with lower ESE who could benefit from more attention. Furthermore, this study is important to optimize interventions targeting ESE.

## Methods

This study is part of a multi-center randomized controlled trial, Act-Active, that evaluates the added value of a behaviorally focused intervention on physical activity, physical fitness and health among persons with subacute SCI (Trial registration**:** NTR2424). Persons aged 18 to 65 years with subacute SCI were recruited from four Dutch rehabilitation centers: Rijndam in Rotterdam, Adelante in Hoensbroek, Heliomare in Wijk aan Zee and De Hoogstraat in Utrecht. To meet inclusion criteria, persons had to be involved in initial inpatient rehabilitation following SCI, (mainly) dependent on a manual wheelchair, able to handcycle, and sufficiently comprehend the Dutch language. Persons were excluded for progressive disease or severe psychiatric condition that could interfere with participation. All participants provided written informed consent. The study was approved by the Medical Ethics Committee of the Erasmus Medical Center in Rotterdam and local approval was granted by the four participating centers. Participants were screened for contraindications to exercise by a rehabilitation physician and all participants completed the Physical Activity Readiness Questionnaire [19].

Data were collected between January 2011 and August 2013 at the four centers using consistent testing protocols. In the current study, baseline data of the longitudinal study that were collected previous to the start of the interventions of Act-Active, two months before discharge from inpatient rehabilitation, were analysed. At this time, all persons with SCI meeting the inclusion criteria were participating in active inpatient rehabilitation. The measurement date was determined based on the discharge date set by the rehabilitation physician. Furthermore, at 2 months before discharge there is still time to start an intervention targeting ESE. A previous study showed that in The Netherlands persons with SCI in rehabilitation spend on average 4.5 h a week in physical therapy, occupational therapy and sports therapy and that therapy programs are more or less similar in different rehabilitation centers [20].

ESE was assessed using the Dutch Exercise Self-Efficacy Scale (ESES) [21]. The ESES consists of 10 items about self-confidence level with respect to performing exercise and daily physical activities. The ESES has demonstrated reliability and validity for use in persons with SCI [21, 22]. The ESES minimum score is 10 and the maximum score is 40, with higher scores indicating higher ESE.

Gender and age of the participants were recorded and lesion level, categorized as paraplegia or tetraplegia, and motor completeness were determined by a rehabilitation physician using international standards [23]. We defined persons 50 years or older as an older person and grouped participants into two age groups, <50 years and ≥50 years. Tetraplegia was defined as a lesion at or above the Thoracic 1 segment, and paraplegia as a lesion below Thoracic 1. Motor completeness included American Spinal Injury Association Impairment Scale categories A and B, whereas motor incompleteness included categories C and D.

**Fig. 1:**
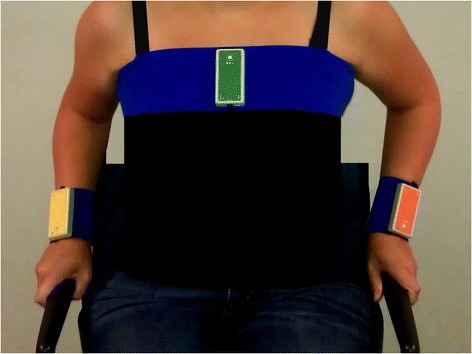
VitaMove Activity Monitor

Physical behavior was objectively measured using the VitaMove activity monitor (2 M Engineering, Veldhoven, The Netherlands) (Fig. 1), an ambulatory monitoring system with body-fixed accelerometers (Freescale MMA7260Q, Denver, USA) [24–26]. This activity monitor was validated for wheelchair-users [24, 26]. The Vitamove consists of three recorders which are wirelessly connected and synchronized every 10 s. One recorder was attached to the sternum and one recorder to each wrist using specially developed belts. The activity monitor was worn continuously for 96 consecutive hours on four weekdays, except during swimming, bathing and sleeping. Due to logistic and technical reasons, the measurement duration goal of 96 h was not always met; the minimum required duration was 24 h for inclusion in analysis. To avoid measurement bias, participants were instructed to continue their ordinary daily routine, including therapies. The principles of the activity monitor were explained only after participants completed the randomized controlled trial. Measurements were uploaded to a computer for kinematic analysis using VitaScore Software (VitaScore BV, Gemert, The Netherlands). A detailed description of this configuration and analysis has been described elsewhere [25, 26]. Every second of the measurement was assigned to one of the four categories: sitting, lying, wheelchair propulsion and handcycling. The following outcome measures were determined as a mean of available measurement days:

- Duration of wheeled physical activity, including wheelchair propulsion and hand cycling, in hours per 24-hour period.
- Duration of sedentary daytime bouts longer than 30 min during day time, including sitting and lying without interruption by physical activity for a minimum of 5 s, in hours, per 24-hour period.
- Mean motility per 24-hour period. Motility is based on the variability of the accelerometer signal of the trunk and arm recorders and is a measure of intensity and duration of all movement, expressed in gravitational force (g).

Physical capacity was determined during a maximal handcycle test using a Tacx Flow ergotrainer (Tacx, The Netherlands and Double Performance, The Netherlands). The test was performed in an add-on handcycle, often used by the participant during rehabilitation and provided by the rehabilitation center. The test started with a warm-up period of 3 min during which the participants cycled on the resistance equal to the resistance of the first minute of the handcycle test. The resistance was estimated based on lesion characteristics and the warm-up period was used to check if the estimation seemed to be correct such that duration of the test would be between 8–12 min. After this warm-up period there was sufficient time to rest. During the test, the resistance was increased every minute by 2 to 10 W, depending on lesion characteristics. Throughout the test, participants cycled at a cadence of 60 rpm. The test ended when the participant stopped voluntarily due to exhaustion, or when the participant was unable to maintain the target cadence. During the test, oxygen uptake (VO_2_) was measured using an Oxycon (Jaeger, Germany). VO_2_peak was defined as the highest mean oxygen uptake during 30 s and expressed in liters per minute (L/min). Furthermore, power was measured continuously with the Tacx Flow ergotrainer. When using correction equations, this ergotrainer has been found reliable and valid in estimating power [27]. Peak power output (POpeak, in Watt) was defined as the highest power output sustained for a minimum of 30 s.

### Statistical analyses

Shapiro-Wilk test showed that ESE data were not normally distributed (W(37) = 0.90, *p* < 0.01). Differences in ESE between subgroups men/women, <50 years/ ≥50 years, tetraplegia /paraplegia, and motor complete/motor incomplete were assessed with Mann-Whitney tests. The statistical significance level was set at *p* ≤ 0.05. Spearman correlations were used to assess the relation between ESE and wheeled physical activity, sedentary daytime bouts longer than 30 min, motility, PO_peak_ and VO_2_peak in the total group. A correlation between 0.3–0.49 was defined as a moderate linear relation and 0.5–1.0 as a strong relation [28]. For outcomes with moderate and strong relations, spearman correlations were determined separately for the subgroups with tetraplegia and paraplegia and presented in scatter plots. Statistical analyses were performed using SPSS 20 (SPSS Inc, Chicago, IL, USA).

## Results

A total of 45 persons with SCI agreed to participate. ESE data were not available for eight participants, thus the present study included ESE data of 37 participants. ESE data were missing in two persons due to sickness of a research assistant and in six persons because they did not fill out the questionnaire. Data of the activity monitor were missing for two persons because these measurements did not meet the minimum required duration. Nine participants were unable to perform the maximal handcycle test due to contraindications for maximal exercise. Of an additional four participants, who did have POpeak data available, VO_2_ data were unavailable: two because of technical problems and two because of bacterial infections. Table 1 shows participant characteristics.

**Table 1:** Participant characteristics (*n* = 37)

Age, median (IQR)	44 (30-56)
Men, n (%)	32 (86)
Tetraplegia/paraplegia, n (%) [range]	
Tetraplegia	12 (32) [C5–Th1]
Paraplegia	25 (68) [Th2–L3]
AIS, n (%)	
Motor incomplete	13 (35)
Motor complete	24 (65)
Time since injury, median days (IQR)	124 (89–160)
Time in rehabilitation, median days (IQR)	83 (57–125)
Cause of injury, n (%)	
Traumatic	26 (70)
Non-traumatic	9 (24)
Unknown	2 (5)
Wheeled physical activity (in hours/24-hour period), median (IQR), *n* = 35	1.12 (0.80–1.58)
Sedentary day time >30 min. (in hours/24-hour period), median (IQR), *n* = 35	1.55 (0.90–3.14)
Motility (in g), median (IQR), *n* = 35	16.20 (13.00–20.05)
VO_2peak_ (in L/min), median (IQR), *n* = 24	1.15 (0.92–1.62)
PO_peak_ (in Watt), median (IQR), *n* = 28	50.37 (27.14–69.24)

IQR = interquartile range

Median ESE was 38.0 (IQR = 32.0–38.5) for women and 36.0 (IQR = 34.0–39.0) for men. Our sample included only five women, all of whom had motor complete paraplegia. Because the number of women was small and lesions were uniform, we did not assess ESE in subgroups based on gender.

Table 2 shows median values on ESE for the total group and for subgroups based on age and lesion characteristics. Compared to persons with paraplegia, persons with tetraplegia seemed to have lower ESE(Z = −1.93, *p* = 0.054).

**Table 2:** Exercise self-efficacy for the total group and for subgroups

	ESE Median (IQR)	Subgroup analysis
All, *n* = 37	37.0 (34.0–39.0)	
Age		
< 50 years, *n* = 25	37.0 (34.0–39.0)	Z = −0.10, *p* = 0.92
≥ 50 year, *n* = 12	36.5 (32.5–39.0)	
Tetraplegia/Paraplegia		
Tetraplegia, *n* = 12	35.0 (31.8–37.0)	Z = −1.93, *p* = 0.05
Paraplegia, *n* = 25	38.0 (34.5–39.5)	
Completeness		
Motor incomplete, *n* = 13	35.0 (32.5–38.5)	Z = −0.91, *p* = 0.36
Motor complete, *n* = 24	37.0 (34.0–39.0)	

**Fig. 2:**
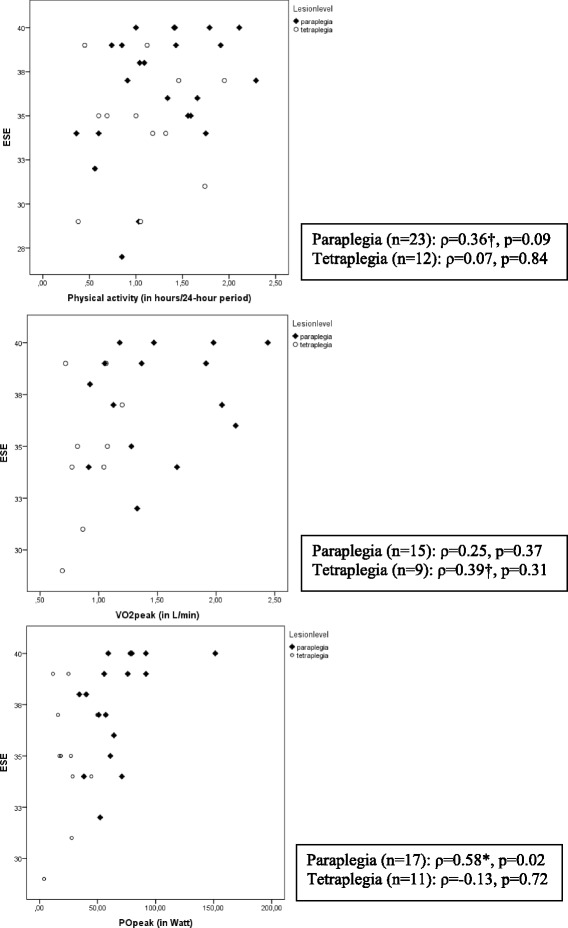
Relations between ESE and physical activity and physical capacity in subgroups based on lesion characteristics, † indicates moderate relation, *strong relation

In Table 3 the linear relations between ESE and physical behavior and physical capacity are reported. For the total group, ESE showed a moderate relation with VO_2peak_ (*ρ* = 0.45, *p* = 0.03) and a strong relation with PO_peak_ (*ρ* = 0.52, *p* < 0.01). The relation of ESE with physical activity was moderate: *ρ* = 0.31, *p* = 0.07. Figure 2 describes the relations separately for persons with paraplegia and tetraplegia for physical activity, VO_2peak_ and PO_peak_. In persons with paraplegia a strong relation was found for ESE with PO_peak_ (*ρ* = 0.58, *p* = 0.02). Furthermore, the relation of ESE with physical activity for persons with paraplegia was moderate *ρ* = 0.36, *p* = 0.09. For persons with tetraplegia, a moderate relation was found between ESE and VO_2peak_: *ρ* = 0.39, *p* = 0.31.

**Table 3:** Relations between ESE and physical activity and physical capacity

	*n*	ESE	
		*ρ* ^c^	*p*
Physical activity (in hours/24-hour period)	35	0.31^a^	0.07
Sedentary day time >30 min (in hours/24-hour period)	35	−0.06	0.73
Motility (in g)	35	0.12	0.51
VO_2peak_ (in L/min)	24	0.45^a^	0.03
PO_peak_ (in Watt)	28	0.52^b^	<0.01

*ρ* ^c^ = Spearmans’ Rho

^a^moderate relation ^b^strong relation

## Discussion

This study assessed ESE in persons with subacute SCI dependent on a manual wheelchair. Lesion level when dichotomized to paraplegia and tetraplegia demonstrated a significant difference between groups in favor of persons with paraplegia when ESE was considered. No significant between group differences were noted when age was categorized as <50 and ≥50 or motor completeness categories were examined. A moderate linear relationship was noted between ESE and physical activity and a strong relationship between ESE and physical capacity in persons with paraplegia. A moderate linear relationship was noted between ESE and VO_2peak_ in persons with tetraplegia.

Generally, ESE seemed relatively high in our group of persons with subacute SCI, median ESE was 37 with a maximum score of 40. However, previous studies using the ESES in persons with SCI in the chronic phase and persons with multiple sclerosis also found relatively high median scores [29, 30]. Nevertheless, the range in ESE (27 to 40) was large enough to test for differences between subgroups in ESE and to assess relations with physical behavior and physical capacity. It should be noted that after rehabilitation ESE possibly changes. In the home situation persons are no longer participating in a program which includes physical activity, but they have to initiate it themselves. Further longitudinal studies are of interest.

Lower ESE in persons with tetraplegia indicates that that this subgroup can benefit from extra attention in the promotion of physical activity and exercise, e.g. by offering them extra sessions in behavioral interventions targeting ESE. This result is in line with previous findings that persons with tetraplegia are less physically active and have less physical capacity in the year following discharge than persons with paraplegia [31, 32]. Since persons with tetraplegia have greater barriers to overcome in order to be physically active, this also should be an important aspect in interventions promoting physical activity. For example by learning to and practicing how to use a handcycle for daily activities or informing about sports activities as swimming and wheelchair rugby. Results were not in line with another previous study in which no significant differences in ESE were found between persons with subacute tetraplegia and paraplegia [33]. This is possibly explained by the use of a different questionnaire for exercise self-efficacy assessing more specific constructs, e.g. confidence to engage in moderate and heavy intensity aerobic and strengthening activity for 10, 20, 30, 45 and 60 min without stopping. Further studies on ESE constructs are necessary.

To our knowledge this is the first study assessing relations of ESE with objectively measured physical behavior and physical capacity. Persons with paraplegia with higher PO_peak_ tended to have higher ESE. This indicates that persons who can do more have greater belief in their ability to exercise. Along the similar line, for persons with tetraplegia, a moderate relation was found between ESE and VO_2peak_. However, this analysis was restricted to nine persons and therefore has to be interpreted with caution. Furthermore, persons with paraplegia with higher daily physical activity levels tended to report higher ESE. This indicates that persons who do more might have greater belief in their ability to exercise. However, these results were not supported by the other physical behaviour and physical capacity outcomes. In addition, it should be noted that physical capacity in this phase might be influenced by the physical capacity and physical activity levels before the SCI. Further studies are necessary.

While no differences were found when ESE was compared within categories of completeness and age, ambulatory persons were excluded from the current study and our sample was relatively young with only 12 persons older than 50 and a maximum age of 65 years. Although sample characteristics were similar to those reported in previous Dutch studies [34], our conclusions are limited to persons who are wheelchair dependent, within the age range of 18 to 65 years. Further study in ambulatory persons and those aged above 65 years is necessary.

Unfortunately, we were unable to assess the relation of gender with ESE because our sample included only five women. Previous research among able-bodied found a relation between gender and ESE [15, 16]. However, a previous study among persons with SCI showed ESE to be similar between men and women [21]. This seems in line with the current study, although the number of women was limited.

### Study limitations

Our study was limited by its cross-sectional design, small sample size, and inclusion of few women. Besides, the measurement with the activity monitor was limited to wheeled physical activity. Other types of physical activity and exercise, such as swimming, were not measured. In addition, it should be noted that sedentary time is difficult to classify for persons who are dependent on a manual wheelchair and are not able to stand or walk. Further study of the effectiveness of interventions targeting ESE in persons with subacute SCI is also needed. In addition, studies on ESE and physical activity in ambulatory persons with SCI are of interest.

## Conclusions

In persons with SCI who are dependent on a manual wheelchair, lesion level when categorized as paraplegic and tetraplegic affected ESE whereas age categories and completeness categories did not. Persons with tetraplegia were found to have less confidence with regard to physical activity and exercise indicating that this subgroup can benefit from extra attention in the promotion of physical activity and exercise, e.g. by offering them extra sessions in behavioral interventions targeting ESE. Results in persons with paraplegia indicate that persons who can do more, have greater belief in their ability to exercise. Although less profound this might apply to persons with tetraplegia as well. Besides, for persons with paraplegia results also seem to indicate that persons who actually do more, have greater belief in their ability to exercise. Assessing ESE in the individual might help practitioners in finding the appropriate guidance a person needs for exercise promotion.

## Abbreviations

ESE: Exercise self-efficacy
PO: Power output
SCI: Spinal cord injury
VO: Oxygen uptake

## References

[1] Nandoe Tewarie RD, Hurtado A, Bartels RH, Grotenhuis JA, Oudega M (2010). A clinical perspective of spinal cord injury. NeuroRehabilitation.

[2] Fernhall B, Heffernan K, Jae SY, Hedrick B (2008). Health implications of physical activity in individuals with spinal cord injury: a literature review. J Health Hum Serv Adm.

[3] Nooijen CF, de Groot S, Postma K, Bergen MP, Stam HJ, Bussmann JB (2012). A more active lifestyle in persons with a recent spinal cord injury benefits physical fitness and health. Spinal Cord.

[4] de Groot S, Dallmeijer AJ, Post MW, Angenot EL, van der Woude LH (2008). The longitudinal relationship between lipid profile and physical capacity in persons with a recent spinal cord injury. Spinal Cord.

[5] Haisma JA, Post MW, van der Woude LH, Stam HJ, Bergen MP, Sluis TA (2008). Functional independence and health-related functional status following spinal cord injury: a prospective study of the association with physical capacity. J Rehabil Med.

[6] Simmons OL, Kressler J, Nash MS (2014). Reference Fitness Values in the Untrained Spinal Cord Injury Population. Arch Phys Med Rehabil.

[7] Janssen TW, Dallmeijer AJ, Veeger DJ, van der Woude LH (2002). Normative values and determinants of physical capacity in individuals with spinal cord injury. J Rehabil Res Dev.

[8] Healy GN, Dunstan DW, Salmon J, Cerin E, Shaw JE, Zimmet PZ (2008). Breaks in sedentary time: beneficial associations with metabolic risk. Diabetes Care.

[9] Manns PJ, Dunstan DW, Owen N, Healy GN (2012). Addressing the nonexercise part of the activity continuum: a more realistic and achievable approach to activity programming for adults with mobility disability?. Phys Ther.

[10] Bussmann JB, van den Berg-Emons RJ. To total amount of activity. and beyond: perspectives on measuring physical behavior. Front Psychol. 2013;4:463. 10.3389/fpsyg.2013.00463 PMC3717476 23885248

[11] Ashford S, Edmunds J, French DP (2010). What is the best way to change self-efficacy to promote lifestyle and recreational physical activity? A systematic review with meta-analysis. Br J Health Psychol.

[12] Haskell WL, Lee IM, Pate RR, Powell KE, Blair SN, Franklin BA (2007). Physical activity and public health: updated recommendation for adults from the American College of Sports Medicine and the American Heart Association. Med Sci Sports Exerc.

[13] Fletcher JS, Banasik JL (2001). Exercise self-efficacy. Clin Excell Nurse Pract.

[14] Froehlich-Grobe K, Lee J, Aaronson L, Nary DE, Washburn RA, Little TD (2013). Exercise for Everyone: A randomized controlled trial of Project Workout On Wheels in promoting exercise among wheelchair users. Arch Phys Med Rehabil.

[15] Spence JC, Blanchard CM, Clark M, Plotnikoff RC, Storey KE, McCargar L (2010). The role of self-efficacy in explaining gender differences in physical activity among adolescents: a multilevel analysis. J Phys Act Health.

[16] Clark DO, Nothwehr F (1999). Exercise self-efficacy and its correlates among socioeconomically disadvantaged older adults. Health Educ Behav.

[17] Lapier TK, Cleary K, Kidd J (2009). Exercise self-efficacy, habitual physical activity, and fear of falling in patients with coronary heart disease. Cardiopulm Phys Ther J.

[18] Imayama I, Alfano CM, Mason CE, Wang C, Xiao L, Duggan C (2013). Exercise adherence, cardiopulmonary fitness and anthropometric changes improve exercise self-efficacy and health-related quality of life. J Phys Act Health.

[19] Cardinal BJ, Esters J, Cardinal MK (1996). Evaluation of the revised physical activity readiness questionnaire in older adults. Med Sci Sports Exerc.

[20] van Langeveld SA, Post MW, van Asbeck FW, ter Horst P, Leenders J, Postma K (2011). Contents of physical therapy, occupational therapy, and sports therapy sessions for patients with a spinal cord injury in three Dutch rehabilitation centres. Disabil Rehabil.

[21] Nooijen CF, Post MW, Spijkerman DC, Bergen MP, Stam HJ, van den Berg-Emons RJ (2013). Exercise self-efficacy in persons with spinal cord injury: psychometric properties of the Dutch translation of the Exercise Self-Efficacy Scale. J Rehabil Med.

[22] Kroll T, Kehn M, Ho PS, Groah S (2007). The SCI Exercise Self-Efficacy Scale (ESES): development and psychometric properties. Int J Behav Nutr Phys Act..

[23] Kirshblum SC, Burns SP, Biering-Sorensen F, Donovan W, Graves DE, Jha A (2011). International standards for neurological classification of spinal cord injury (revised 2011). J Spinal Cord Med.

[24] Postma K, van den Berg-Emons HJ, Bussmann JB, Sluis TA, Bergen MP, Stam HJ (2005). Validity of the detection of wheelchair propulsion as measured with an Activity Monitor in patients with spinal cord injury. Spinal Cord.

[25] Bussmann JB, Martens WL, Tulen JH, Schasfoort FC, van den Berg-Emons HJ, Stam HJ (2001). Measuring daily behavior using ambulatory accelerometry: the Activity Monitor. Behav Res Methods Instrum Comput.

[26] Nooijen C, de Groot JF, Stam HJ, van den Berg-Emons R, Bussmann H (2015). Validation of an activity monitor for children who are partly or completely wheelchair-dependent. J Neuroeng Rehabil.

[27] Nooijen CF, Dubbelman P, ter Horst P, Broeksteeg R, Valent LJ, van den Berg-Emons RJ. De reproduceerbaarheid en nauwkeurigheid van de meting van het vermogen: Het inzetten van een ergotrainer voor handbike training en testen. Nederlands Tijdschrift voor Revalidatiegeneeskunde. 2013;6

[28] Cohen J. Applied Multiple Regression/correlation Analyais for the Behavioral Sciences. Taylor & Francis. 2003.

[29] Kroll T, Kratz A, Kehn M, Jensen MP, Groah S, Ljungberg IH (2012). Perceived exercise self-efficacy as a predictor of exercise behavior in individuals aging with spinal cord injury. Am J Phys Med Rehabil.

[30] Stroud N, Minahan C, Sabapathy S (2009). The perceived benefits and barriers to exercise participation in persons with multiple sclerosis. Disabil Rehabil.

[31] van den Berg-Emons RJ, Bussmann JB, Haisma JA, Sluis TA, van der Woude LH, Bergen MP (2008). A prospective study on physical activity levels after spinal cord injury during inpatient rehabilitation and the year after discharge. Arch Phys Med Rehabil.

[32] Haisma JA, Bussmann JB, Stam HJ, Sluis TA, Bergen MP, Dallmeijer AJ (2006). Changes in physical capacity during and after inpatient rehabilitation in subjects with a spinal cord injury. Arch Phys Med Rehabil.

[33] Pelletier CA, Jones G, Latimer-Cheung AE, Warburton DE, Hicks AL (2013). Aerobic Capacity, Orthostatic Tolerance, and Exercise Perceptions at Discharge From Inpatient Spinal Cord Injury Rehabilitation. Arch Phys Med Rehab.

[34] de Groot S, Dallmeijer AJ, Post MW, van Asbeck FW, Nene AV, Angenot EL (2006). Demographics of the Dutch multicenter prospective cohort study 'Restoration of mobility in spinal cord injury rehabilitation'. Spinal Cord.

